# Information spectra and optimal background states for dynamical networks

**DOI:** 10.1038/s41598-018-34528-y

**Published:** 2018-11-01

**Authors:** Delsin Menolascino, ShiNung Ching

**Affiliations:** 1 Department of Electrical and Systems Engineering, Washington University in St. Louis, St. Louis, MO 63130 USA; 2 Department of Biology and Biomedical Sciences, Washington University in St. Louis, St. Louis, MO 63130 USA

## Abstract

We consider the notion of stimulus representation over dynamic networks, wherein the network states encode information about the identify of an afferent input (i.e. stimulus). Our goal is to understand how the structure and temporal dynamics of networks support information processing. In particular, we conduct a theoretical study to reveal how the background or ‘default’ state of a network with linear dynamics allows it to best promote discrimination over a continuum of stimuli. Our principal contribution is the derivation of a matrix whose spectrum (eigenvalues) quantify the extent to which the state of a network encodes its inputs. This measure, based on the notion of a Fisher linear discriminant, is relativistic in the sense that it provides an information value quantifying the ‘knowablility’ of an input based on its projection onto the background state. We subsequently optimize the background state and highlight its relationship to underlying state noise covariance. This result demonstrates how the best idle state of a network may be informed by its structure and dynamics. Further, we relate the proposed information spectrum to the controllabilty gramian matrix, establishing a link between fundamental control-theoretic network analysis and information processing.

## Introduction

In network science, considerable effort has been directed at structural analysis that reveals the interconnection architecture of engineered and biological networks^1–6^. While such analysis can illuminate intriguing and common architectural principles of complex systems, it alone cannot tell us the functionality of such architecture. In other words, to what end is the revealed structure useful? Our goal in this work is to analyze the relationship between structure, dynamics and function of (networked) systems. The specific notion of function that we consider is information coding, which has to do with how networks represent a stimulus or extrinsic input in a way that is useful for downstream processing (i.e., so that an agent can decode the identity of input stimuli based on a ‘read out’ of the state of the network). This sort of coding has been a topic of much interest in theoretical neuroscience, where understanding how networks of neurons represent stimuli is a foundational question^7–9^.

Of course, many general principles of information coding are known from communication theory^10^. However, it is not clear how principles of information transmission, coding/decoding and capacity are impacted when enacted over a networked system, especially one with continuous time dynamics. That is, what structure and dynamical aspects of a network make it a good information encoder? To this end, we principally address two questions: 1) What sorts of dynamics shape the input/output relationship of a network in a way which is effective in the Shannon sense (i.e. some, but not too much, redundancy to enable robust, efficient communication in the presence of noise)? It is especially unclear whether dynamical networks that do a good job encoding and/or processing information are also those that are most *responsive* to their inputs in a control-theoretic sense. Hence the second question: 2) Is a network that is easily controlled by its inputs necessarily one that also effectively encodes information about those inputs? These two related questions constitute the primary focus of the paper.

We consider information processing defined in terms of the extent to which network states/outputs encode their respective inputs. Our particular focus is on the background state of a network and its ability to facilitate information extraction regarding other afferent inputs. Non-zero background states are frequently observed in natural dynamical systems. For example, in the study of brain networks the existence of a ‘resting state’ is well-established experimentally^11–14^. Our goal is to provide a theoretical framework with which we can better understand how non-zero resting states confer *informational* utility. Specifically, we will derive a background state that is optimal according to a novel information measure (also herein derived). In mathematical terms, suppose that a stimulus **u** induces a network state **x**_**u**_. We will quantify the ‘knowability’ of **u** by comparing **x**_**u**_ against a reference background state **x**_ref_. The optimal **x**_ref_ can be interpreted as a ‘state of readiness’ at which the network may be sustained in preparation for activity to follow.

**Figure 1:**
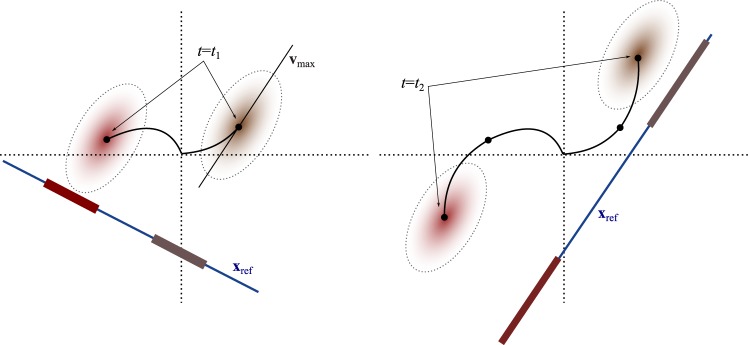
The optimal background state **x**_ref_ amounts to a Fisher linear discriminant, onto which state distributions (induced by inputs) are projected. In the case of Gaussian noise, uncertainty can be visualized in terms of ellipsoids (with principal axis **v**_max_) about the mean. Since the networks are dynamic, the optimal **x**_ref_ will vary with time as the dynamics carry the states forward.

The information measure we employ is based upon the inner product 〈**x**_ref_, **x**_**u**_〉, and is rooted in the method of Fisher linear discriminants^15–17^. This inner product, as is well-known, prescribes the projection of **x**_**u**_ onto **x**_ref_. For vectors of known magnitude, this projection gauges angular separation. Thus, essential characteristics of **x**_**u**_ (and by extension, of **u**) can be gleaned in an easily codified and quantified manner. The potential informational value to be derived from a projection of **x**_**u**_ onto **x**_ref_, depends critically on the effective choice of the background state **x**_ref_ and uncertainty/noise. The choice of **x**_ref_, in turn, depends largely on how a network responds to its inputs as a function of time (see Fig. 1). Noise and uncertainty, likewise, are impacted by the network dynamics.

The formulation of a continuous-time dynamical (networked) system with afferent input is fundamentally aligned with analysis from control theory. A key aspect of our results will be the derivation of a Fisher information matrix, ${ {\mathcal I} }_{{\bf{u}}}$, associated with the above inner product. As we will see, the spectrum of ${ {\mathcal I} }_{{\bf{u}}}$ quantifies the extent to which different afferent inputs produce different state representations. It turns out that this information spectrum has a particular statistical relationship with a traditional element from control theory, the controllability gramian matrix^18^. This is perhaps intuitive since the control gramian is mathematically equivalent to the covariance of a network in response to white noise, a key source of uncertainty (and, thus, information loss). We will formalize this relationship in our results.

Assessment of information propagation through noisy networks has been a topic of increasing interest, and while there are many contexts for which such analyses are relevant, quantifying the information-carrying capacity of (real and/or artificial) neural networks has been an especially active research area^8,9,19,20^. For example, in Zylberberg *et al*.^9^, linear Fisher information is evaluated for a two-layer feedforward network in which a scalar signal is distributed to first-layer nodes and then propagated to the second layer via a weighted matrix, with noise corrupting the output of both layers. The amount of information available about the stimulus, they observe, is dependent on the noise covariance structure at each layer, and on how these covariances relate to one another and to the direction of signal propagation (i.e. the tuning curve). However, the network considered in this work is static, so that input-output relationships are fully determined by network structure alone (i.e. there is no recurrent modulation of the signal, though noise does play a role, obviously).

In contrast, Ganguli *et al*.^8^ quantify the stimulus-encoding capacity of a linear dynamical network, again employing linear Fisher information theory. Here, the stimulus is presented as a pulse at a specific time, the ‘memory trace’ of which is preserved by the network over time to an extent depending on the network’s topology, and the statistical behavior of state noise.

Our work employs the dynamical framework of Ganguli *et al*.^8^, while considering multi-variate stimuli, akin to the ‘tuning curves’ of Zylberberg *et al*.^9^ In fact our framework allows for stimuli of arbitrary dimensions, although here we do constrain the inputs (stimuli) to be constant (for reasons addressed in the Discussion). Also our notion of *how* stimulus information is encoded is different. Specifically, we employ the inner-product based readout, facilitating a comparison between an output vector **x**_**u**_ and a reference **x**_ref_, as mentioned above.

## Results

### Problem Formulation and Preliminaries

#### Linear Dynamical Networks

Linear dynamics have been used to describe complex networks in several contexts^21–23^, with the caveat that such dynamics provide only local approximations of more complex, nonlinear regimes. Proceeding with this limitation in mind, we consider a linear dynamical system (network) with noise, of the form:

1 $$\dot{{\bf{x}}}(t)={\bf{A}}{\bf{x}}(t)+{\bf{B}}{\bf{u}}+{\bf{w}}(t)$$

where the *n*-dimensional state vector **x**’s recurrent dynamics are described by adjacency matrix ${\bf{A}}\in {{\mathbb{R}}}^{n{\rm{x}}n}$, input matrix ${\bf{B}}\in {{\mathbb{R}}}^{n{\rm{x}}m}$ mediating the *m*-dimensional input **u**, taken here to be constant (see Discussion), and zero-mean gaussian noise **w**(*t*), which has covariance matrix Σ_**w**_. We point out the fact that the term dynamical network is used here to imply time-evolution in the network states, as opposed to a time-varying vector field; that is, **A** is constant. We wish to consider the linear Fisher information regarding **u** given the inner product of the state **x**(*t*) (which varies in time) and a reference background **x**_ref_. By basic linear system theory

2 $${\bf{x}}(t)\sim {\mathscr{N}}({\int }_{0}^{t}\,{{\rm{e}}}^{{\bf{A}}(t-\tau )}{\rm{d}}\tau {\bf{B}}{\bf{u}},{{\rm{\Sigma }}}_{x(t)}),$$

where ${{\rm{\Sigma }}}_{{\bf{x}}(t)}\in {{\mathbb{R}}}^{n\times n}$ is a covariance matrix determined by the system dynamics.

#### Inner Product and Fisher Information

As we seek to quantify the extent to which the inner product of **x**(*t*) and **x**_ref_ encodes information about the input **u** giving rise to **x**(*t*), we employ the Fisher information matrix, denoting it ${ {\mathcal I} }_{{\bf{u}}}$, which is given by

3 $${ {\mathcal I} }_{{\bf{u}}}(t)=\frac{\partial {({\mathbb{E}}[\langle {\bf{x}}({\rm{t}}),{{\bf{x}}}_{{\rm{ref}}}\rangle ])}^{T}}{\partial {\bf{u}}}{{\rm{\Sigma }}}_{\langle {\bf{x}}(t),{{\bf{x}}}_{{\rm{ref}}}\rangle }^{-1}\frac{\partial {\mathbb{E}}[\langle {\bf{x}}({\rm{t}}),{{\bf{x}}}_{{\rm{ref}}}\rangle ]}{\partial {\bf{u}}}$$

where 〈**x**, **x**_ref_〉 = **x**^*T*^**x**_ref_ and ${{\rm{\Sigma }}}_{\langle {\bf{x}},{{\bf{x}}}_{{\rm{ref}}}\rangle }$ denotes the variance of this inner product. The inner product can be interpreted in several ways, including as the correlation or contrast between two competing states. The Fisher information lower bounds the variance of an estimate of **u** based on measurement of the inner product.

From (3), and taking into account the independence of **x** and **x**_ref_ we have

4 $${ {\mathcal I} }_{{\bf{u}}}=\frac{\partial {({\mathbb{E}}{[{\bf{x}}]}^{T}{\mathbb{E}}[{{\bf{x}}}_{{\rm{ref}}}])}^{T}}{\partial {\bf{u}}}{{\rm{\Sigma }}}_{\langle {\bf{x}},{{\bf{x}}}_{{\rm{ref}}}\rangle }^{-1}\frac{\partial {\mathbb{E}}{[{\bf{x}}]}^{T}{\mathbb{E}}[{{\bf{x}}}_{{\rm{ref}}}]}{\partial {\bf{u}}}$$

(where we have dropped dependence on *t* for notational convenience).

Defining

5 $${\rm{\Gamma }}\equiv {\int }_{0}^{t}\,{{\rm{e}}}^{{\bf{A}}(t-\tau )}{\rm{d}}\tau $$

we obtain

6 $${ {\mathcal I} }_{{\bf{u}}}=\frac{\partial {({\mathbb{E}}{[{\bf{x}}]}^{T}{\mathbb{E}}[{{\bf{x}}}_{{\rm{ref}}}])}^{T}}{\partial {\bf{u}}}{{\rm{\Sigma }}}_{\langle {\bf{x}},{{\bf{x}}}_{{\rm{ref}}}\rangle }^{-1}\frac{\partial ({\mathbb{E}}{[{\bf{x}}]}^{T}{\mathbb{E}}[{{\bf{x}}}_{{\rm{ref}}}])}{\partial {\bf{u}}}$$

7 $$=\,\frac{\partial {(({\rm{\Gamma }}{\bf{B}}{\bf{u}}{)}^{T}{{\bf{x}}}_{{\rm{ref}}})}^{T}}{\partial {\bf{u}}}{{\rm{\Sigma }}}_{\langle {\bf{x}},{{\bf{x}}}_{{\rm{ref}}}\rangle }^{-1}\frac{\partial (({\rm{\Gamma }}{\bf{B}}{\bf{u}}{)}^{T}{{\bf{x}}}_{{\rm{ref}}})}{\partial {\bf{u}}}$$

8 $$=\,{{\bf{B}}}^{T}{{\rm{\Gamma }}}^{T}{{\bf{x}}}_{{\rm{ref}}}{{\rm{\Sigma }}}_{\langle {\bf{x}},{{\bf{x}}}_{{\rm{ref}}}\rangle }^{-1}{{\bf{x}}}_{{\rm{ref}}}^{T}{\rm{\Gamma }}{\bf{B}}$$

Using the derivation given explicitly in the Methods section, we obtain the Fisher information matrix

9 $${ {\mathcal I} }_{{\bf{u}}}=\frac{{{\bf{B}}}^{T}{{\rm{\Gamma }}}^{T}{{\bf{x}}}_{{\rm{ref}}}{{\bf{x}}}_{{\rm{ref}}}^{T}{\rm{\Gamma }}{\bf{B}}}{{{\bf{x}}}_{{\rm{ref}}}^{T}{{\rm{\Sigma }}}_{{\bf{x}}}{{\bf{x}}}_{{\rm{ref}}}}$$

where ${{\rm{\Sigma }}}_{{\bf{x}}}$ is the state covariance matrix as introduced in (2).

In seeking a holistic assessment of the matrix ${ {\mathcal I} }_{{\bf{u}}}$, we employ the trace, which is the summed component-wise variance in our estimation of **u**. Since the ${ {\mathcal I} }_{{\bf{u}}}$ is an outer product of two vectors (scaled by the denominator) we may express its trace as their scaled inner product:

10 $${\rm{tr}}({ {\mathcal I} }_{{\bf{u}}}(t))=\frac{{{\bf{x}}}_{{\rm{ref}}}^{T}{\rm{\Gamma }}(t){\bf{B}}{{\bf{B}}}^{T}{\rm{\Gamma }}{(t)}^{T}{{\bf{x}}}_{{\rm{ref}}}}{{{\bf{x}}}_{{\rm{ref}}}^{T}{{\rm{\Sigma }}}_{{\bf{x}}}(t){{\bf{x}}}_{{\rm{ref}}}},$$

where dependence on *t* has again been made explicit.

#### Linear Dynamics and Noise Ellipsoids

Figure 1 provides a schematic of the problem formulation. Because our dynamics are linear, at any given time *t* the state of the network is a Gaussian random vector. The covariance of the state can be used to parameterize a quadratic form whose level sets constitute ellipsoids that encapsulate the mean. We denote the principal eigenvector of the covariance matrix as **v**_max_. These ellipsoids capture the noise-driven uncertainty in the state. As we will soon see, the optimal **x**_ref_ amounts to a Fisher linear discriminant that best disassociates two competing state distributions (ellipsoids), each associated with a different stimulus. As the network dynamics carry these trajectories forward in time, the optimal **x**_ref_ will in general change.

#### Network Parameterization, Actuated Nodes and Steady-State Assumption

We will focus our attention on networks that have a Barabási-Albert (scale-free) topology^24^. The off-diagonal elements of **A** are binary, while the diagonal elements are assigned large enough negative values to ensure stability (see Methods). The dynamics of such networks are asymptotically stable so that in the absence of stimuli and noise, all states return to the origin.

In our analysis we will vary the structure of how inputs impinge on network nodes. In particular, for an *n* node network, only *n*_d_ ≤ *n* nodes will receive input. These actuated nodes are sometimes referred to as ‘driver’ nodes^25–27^. We will mostly consider the case when each actuated node recieves an independent input, so that

11 $${\bf{B}}=[\frac{{{\rm{I}}}_{{n}_{{\rm{d}}}}}{0}]$$

where ${{\rm{I}}}_{{n}_{{\rm{d}}}}$ is the identity matrix of dimension *n*_d_ (the number of driven nodes).

We make the assumption that the noise covariance is always at steady-state. In concept here is that the dynamics of the network are persistently excited by ongoing noise, while receiving stimuli in a temporally punctate manner. To be mathematically precise, under this assumption (2) becomes:

12 $${\bf{x}}(t)\sim {\mathscr{N}}({\int }_{0}^{t}\,{{\rm{e}}}^{{\bf{A}}(t-\tau )}{\rm{d}}\tau {\bf{B}}{\bf{u}},{{\rm{\Sigma }}}_{x(\infty )}),$$

Critically, we assume the pair **A**, **B** is controllable, so that the controllability gramian (precisely defined later) is full-rank. A final important assumption pertains to the specification of *t*. In cases when *t* is assumed to be at steady state, we set *t* = 10 (which we find is five times longer than the time-constant of our considered networks). In other cases, we will vary *t* to assess the role of dynamics.

### An optimal reference state x_ref_ exists, maximizing information about u

We are interested, for the moment, in which choice of **x**_ref_ will maximize (10). That is, we seek to answer the question: Of all possible background states **x**_ref_, which one will provide the most information about a stimulus **u** (with its resultant output **x**), given a readout of the inner product 〈**x**_ref_, **x**〉. In order to find this ‘ideal’ reference stimulus, we transform (10) as follows:

13 $${\rm{tr}}({ {\mathcal I} }_{{\bf{u}}})=\frac{{{\bf{x}}}_{{\rm{ref}}}^{T}{\rm{\Gamma }}{\bf{B}}{{\bf{B}}}^{T}{{\rm{\Gamma }}}^{T}{{\bf{x}}}_{{\rm{ref}}}}{{{\bf{x}}}_{{\rm{ref}}}^{T}{\bf{L}}{{\bf{L}}}^{T}{{\bf{x}}}_{{\rm{ref}}}}$$

where **LL**^*T*^ (**L** is lower-triangular) is the Cholesky decomposition of Σ_**x**_.Continuing, we have

14 $${\rm{tr}}({ {\mathcal I} }_{{\bf{u}}})=\frac{{{\bf{x}}}_{{\rm{ref}}}^{T}{\rm{\Gamma }}{\bf{B}}{{\bf{B}}}^{T}{{\rm{\Gamma }}}^{T}{{\bf{x}}}_{{\rm{ref}}}}{{{\bf{x}}}_{{\rm{ref}}}^{T}{\bf{L}}{{\bf{L}}}^{T}{{\bf{x}}}_{{\rm{ref}}}}$$

15 $$=\,\frac{{{\bf{x}}}_{\ast }^{T}{{\bf{L}}}^{-T}{\rm{\Gamma }}{\bf{B}}{{\bf{B}}}^{T}{{\rm{\Gamma }}}^{T}{{\bf{L}}}^{-1}{{\bf{x}}}_{\ast }}{{{\bf{x}}}_{\ast }^{T}{{\bf{x}}}_{\ast }}$$

where x_*_ = **L**^*T*^**x**_ref_. Therefore, for simplicity of notation letting **S** = **L**^−1^Γ**BB**^*T*^Γ^*T*^**L**^−*T*^, we have the familiar Rayleigh quotient

16 $${\rm{tr}}({ {\mathcal I} }_{{\bf{u}}})=\frac{{{\bf{x}}}_{\ast }^{T}{\bf{S}}{{\bf{x}}}_{\ast }}{{{\bf{x}}}_{\ast }^{T}{{\bf{x}}}_{\ast }}$$

whose values lie in the range ${\lambda }_{{\rm{\min }}}\le {\rm{tr}}({ {\mathcal I} }_{{\bf{u}}})\le {\lambda }_{{\rm{\max }}}$ and which achieves its extrema for ${{\bf{x}}}_{\ast }={{\bf{x}}}_{{\rm{\min }}}^{\ast }$ and ${{\bf{x}}}_{\ast }={{\bf{x}}}_{{\rm{\max }}}^{\ast }$ where ${{\bf{x}}}_{{\rm{\min }}}^{\ast }$ and ${{\bf{x}}}_{{\rm{\max }}}^{\ast }$ are the eigenvectors of **S** associated with eigenvalues *λ*_min_ and *λ*_max_ respectively. We then make the reverse transformation

17 $${{\bf{x}}}_{{\rm{ref}}}={{\bf{L}}}^{-T}{{\bf{x}}}_{{\rm{\max }}}^{\ast }$$

to obtain our ideally contrasting reference state. Mathematically (and as depicted in Fig. 1) **x**_ref_ is in fact the Fisher linear discriminant that best separates the induced state distributions associated with any two randomly chosen inputs.

**Figure 2:**
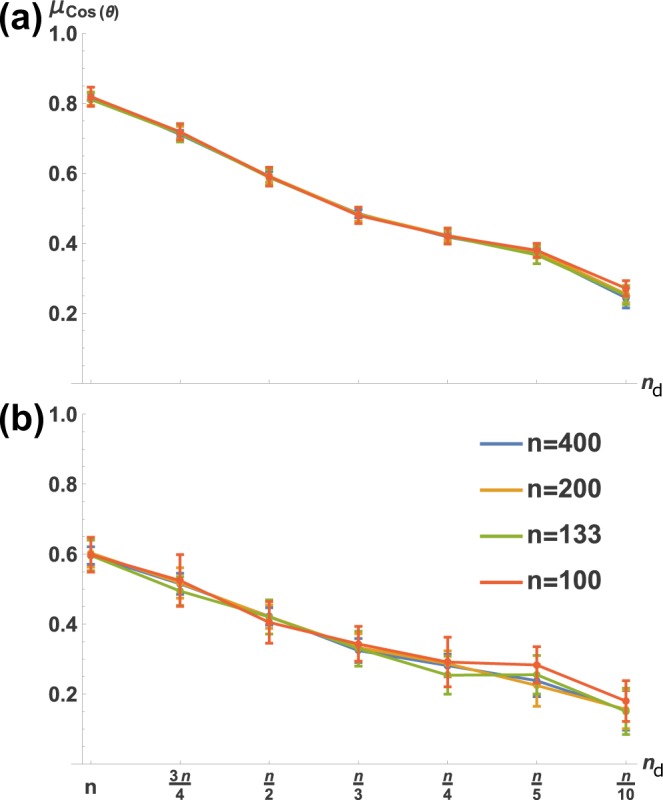
Fidelity of optimally contrasting reference state **x**_ref_ to system noise covariance decreases monotonically with *n*_d_. Shown is how **x**_ref_ aligns with the principal eigenvector (denoted **v**_max_) of noise covariance matrix Σ_**w**_. *μ*_Cos(*θ*)_ is the mean, over 30 network realizations, of the cosine of the angular difference (*θ*) between **x**_ref_ and **v**_max_. Error bars are standard deviations. 30 realizations were evaluated for (**a**) identity and (**b**) random **B** matrices.

**Figure 3:**
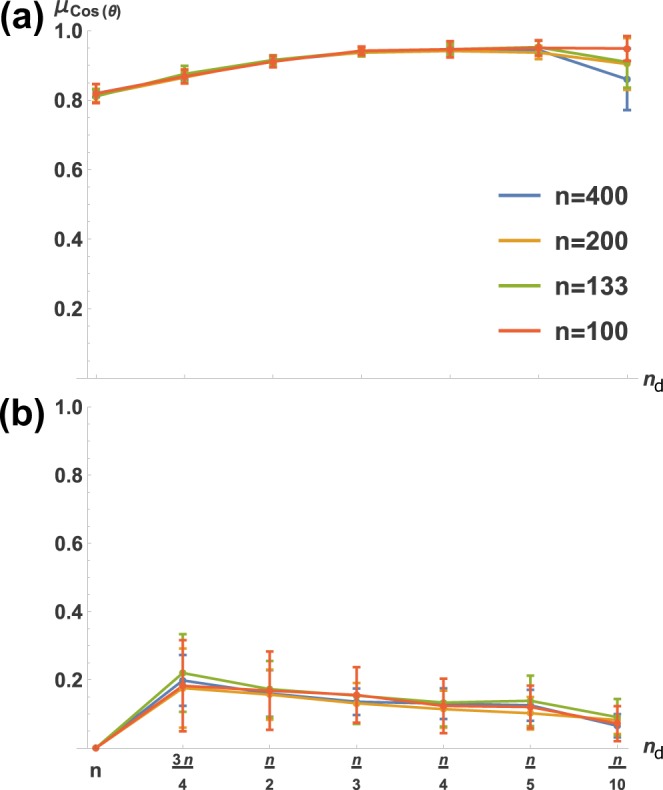
Actuated nodes of the ideal background (**x**_ref_) are ‘required’ to be aligned with noise; non-actuated nodes are not. Shown is alignment of **x**_ref_ with principal noise covariance direction **v**_max_ (as in Fig. 2); here **x**_ref_ and **v**_max_ are partitioned so that (**a**) reflects only actuated and (**b**) only non-actuated nodes. *μ*_Cos(*θ*)_ is as in Fig. 2, again for 30 network realizations.

Previous results^9^ have shown that an optimally informative ‘signal direction’ in a non-dynamical feedforward network is one which align with the principal axis of the noise covariance ellipsoid. Similarly, with our dynamical setup, we decided to explore the optimal **x**_ref_ qualitatively by examining to what extent it aligns with the principal axis of the noise covariance ellipsoid (**v**_max_ of Σ_**x**_ in (10)). The results are shown in Fig. 2. We notice in Fig. 2 that the ideal **x**_ref_ changes its orientation relative to **v**_max_ as a function of *n*_d_. This orientation is virtually uncorrelated with network size and is very predictable, as we ran 30 network realizations for each *n*, *n*_d_ pair and found little variability. We hypothesized that this was due to prioritization of the fidelity of the portion of **x**_ref_ corresponding to actuated nodes, which would explain why relatively under-actuated networks showed greater overall angular divergence between **x**_ref_ and **v**_max_. This is indeed the case, as shown in Fig. 3. We first examined actuated nodes, then non-actuated nodes, by segmenting **x**_ref_ and **v**_max_ into the first *n*_d_ elements (Fig. 3(a)), then the last *n* − *n*_d_ elements (Fig. 3(b)). Clearly, the actuated part of **x**_ref_ is required to be much more similar to the corresponding part of **v**_max_ than is true for the non-actuated part.

**Figure 4:**
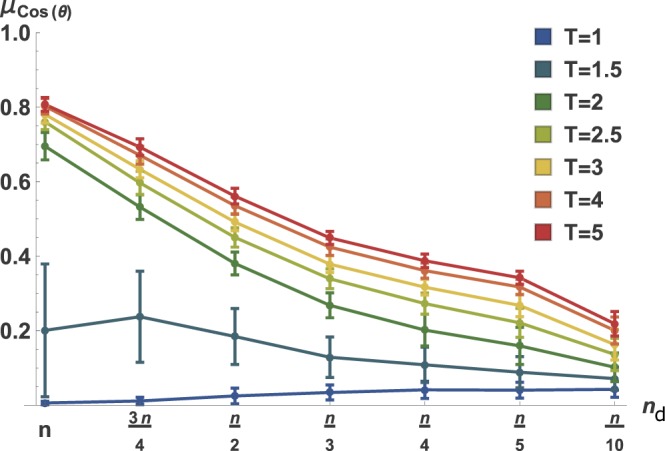
The ideal contrast becomes more aligned with noise covariance as time progresses. Shown is the time-evolution of the relative orientation between the optimally contrasting state **x**_ref_ and the principal noise eigenvector **v**_max_. At lower values of *T*, **x**_ref_ is nearly orthogonal to **v**_max_, while as *T* gets larger, **x**_ref_ becomes much more aligned with **v**_max_, although this alignment approaches a limit, which also varies monotonically with *n*_d_.

Aside from the dependence of the optimal **x**_ref_ on input structure (particularly *n*_d_), we also analyzed how the orientation of **x**_ref_, relative to **v**_max_, changes with time. Since, as mentioned above, we are working in a dynamical regime, a time-dependent analysis is straightforward. To this end, we evaluated the orientation of **x**_ref_, relative to **v**_max_, at several time points, using the same methodology employed above, with the results shown in Fig. 4. We see that the orientation of **x**_ref_ relative to **v**_max_ does indeed change with time, apparently smoothly, and that **x**_ref_ becomes more similar to **v**_max_ as time advances. This is especially true for fully- or nearly fully-actuated networks, but is generally true for all input scenarios.

Thus, the optimally contrasting *background*/*reference state* is fundamentally dependent on the input structure of the network *and* the time evolution of network dynamics.

### An optimal reference input u_ref_ exists, maximizing information about u

We expanded our inquiry to analyze admissible reference inputs **u**_ref_ which could give rise to an optimally contrasting state **x**_ref_. More generally, we asked: Of all possible stimuli, does there exist a best one **u**_ref_, resulting in an output ${\tilde{{\bf{x}}}}_{{\rm{ref}}}$, that provides information about all others. In this formulation, ${\tilde{{\bf{x}}}}_{{\rm{ref}}}$ is not longer unconstrained, but rather is determined by:

18 $${\tilde{{\bf{x}}}}_{{\rm{ref}}}={\rm{\Gamma }}{\bf{B}}{{\bf{u}}}_{{\rm{ref}}}$$

Using (18), we can find the optimal reference stimulus via a similar sequence of steps as in the previous subsection, defining

19 $${\rm{tr}}({ {\mathcal I} }_{{\bf{u}}})=\frac{{{\bf{u}}}_{{\rm{ref}}}^{T}{{\bf{B}}}^{T}{{\rm{\Gamma }}}^{T}{\rm{\Gamma }}{\bf{B}}{{\bf{B}}}^{T}{{\rm{\Gamma }}}^{T}{\rm{\Gamma }}{\bf{B}}{{\bf{u}}}_{{\rm{ref}}}}{{{\bf{u}}}_{{\rm{ref}}}^{T}{{\bf{B}}}^{T}{{\rm{\Gamma }}}^{T}{{\rm{\Sigma }}}_{{\bf{x}}}{\rm{\Gamma }}{\bf{B}}{{\bf{u}}}_{{\rm{ref}}}}$$

20 $$=\,\frac{{{\bf{u}}}_{{\rm{ref}}}^{T}{{\bf{B}}}^{T}{{\rm{\Gamma }}}^{T}{\rm{\Gamma }}{\bf{B}}{{\bf{B}}}^{T}{{\rm{\Gamma }}}^{T}{\rm{\Gamma }}{\bf{B}}{{\bf{u}}}_{{\rm{ref}}}}{{{\bf{u}}}_{{\rm{ref}}}^{T}{\bf{L}}{{\bf{L}}}^{T}{{\bf{u}}}_{{\rm{ref}}}}$$

where **LL**^*T*^ (**L** is lower-triangular) is the Cholesky decomposition of **B**^*T*^Γ^*T*^Σ_**x**_Γ**B**, which is positive-definite (a requirement for this decomposition) since covariance matrix Σ_**x**_ is inherently positive-definite and thus can be Cholesky decomposed into ${{\bf{L}}}_{{\rm{\Sigma }}}{{\bf{L}}}_{{\rm{\Sigma }}}^{T}$, so that the matrix **B**^*T*^Γ^*T*^Σ_**x**_Γ**B** can be written **QQ**^*T*^ for **Q** = **B**^*T*^Γ^*T*^**L**_Σ_ and is thus positive-semidefinite, while the full-rank condition of **Q** ensures positive-definiteness.

Defining **S**_**u**_ ≡ **L**^−*T*^**B**^*T*^Γ^*T*^Γ**BB**^*T*^Γ^*T*^Γ**BL**^−1^, we arrive at

21 $${\rm{tr}}({ {\mathcal I} }_{{\bf{u}}})=\frac{{{\bf{u}}}_{\ast }^{T}{{\bf{S}}}_{{\bf{u}}}{{\bf{u}}}_{\ast }}{{{\bf{u}}}_{\ast }^{T}{{\bf{u}}}_{\ast }}$$

whose values lie in the range ${\lambda }_{{\rm{\min }}}\le {\rm{tr}}({ {\mathcal I} }_{{\bf{u}}})\le {\lambda }_{{\rm{\max }}}$ and which achieves its extrema for ${{\bf{u}}}_{\ast }={{\bf{u}}}_{{\rm{\min }}}^{\ast }$ and ${{\bf{u}}}_{\ast }={{\bf{u}}}_{{\rm{\max }}}^{\ast }$ where ${{\bf{u}}}_{{\rm{\min }}}^{\ast }$ and ${{\bf{u}}}_{{\rm{\max }}}^{\ast }$ are the eigenvectors of **S**_**u**_ associated with eigenvalues *λ*_min_ and *λ*_max_, respectively. We then make the reverse transformation ${{\bf{u}}}_{{\rm{ref}}}={{\bf{L}}}^{-T}{{\bf{u}}}_{{\rm{\max }}}^{\ast }$ to obtain our ideally contrasting reference input.

We pause for a moment to consider the significance of this ‘optimal’ **u**_ref_ (i.e. the eigenvector of **S**_**u**_ which optimizes (21)). The existence of such an optimum means that for a given network, there is one input whose induced state best contrasts those of all other inputs.

### The optimally contrasting input targets specific nodes in a concentrated manner, but not necessarily nodes of highest degree

**Figure 5:**
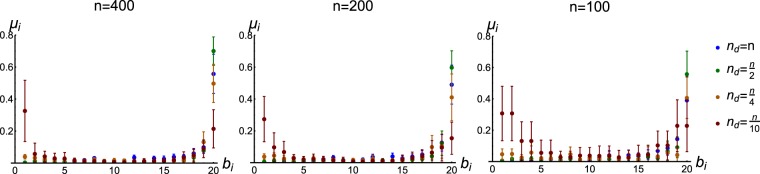
The optimally contrasting input **u**_ref_ targets network ‘hubs’, but to a degree which varies with *n*_d_. 30 networks were realized with for each size (*n*) and driver node (*n*_d_) combination. For a given network size, the graph shows the mean (*μ*_*i*_) of the squared entries of the (normalized) optimal **u**_ref_. The entries of **u**_ref_ are sorted according to the degree of targeted nodes (abscissa is a percentile, binned in increments of 5%, so that each *b*_*i*_ represents 5% of the nodes). Note that when *n* = 100 and ${n}_{{\rm{d}}}=\frac{n}{10}$ there are twice as many bins as controlled nodes, hence the duplicity of values.

We sought to characterize the ‘optimally informative’ **u**_ref_ by examining its entries (recall that we are in the domain of constant inputs) as they relate to the connectivity degree of actuated nodes. Clearly, **u**_ref_ has cardinality *n*_d_ (see (11)). Since, then, there is a one-to-one relationship between the *n*_d_ entries of **u**_ref_ and the driven nodes, we are able to learn about which nodes may be specially ‘targeted’ by an optimally contrasting **u**_ref_. Intuition would suggest that the targeted nodes would simply be the hubs, that is, that the higher the degree of a node, the higher the value of the corresponding entry of **u**_ref_. This is borne out in simulation, but to an extent which varies consistently with network size (*n*) and *n*_d_. Examining Fig. 5, we see that for larger networks wherein all nodes are controlled, nearly all of the large entries of **u**_ref_ are concentrated toward nodes in the top 5% by degree ranking (i.e. the hubs), while as we control fewer nodes, a majority of the large entries are directed toward the hubs, but this majority becomes smaller as *n*_d_ decreases. Also, looking at the different network sizes, we see that, in general, larger networks show a more pronounced ‘targeting’ of the hubs, while in smaller networks the hubs are still targeted but to a lesser extent. It should be pointed out that **u**_ref_ is unitary, meaning there is an essential trade-off between how much energy can be focused on hubs and how much can be focused elsewhere (as is easily seen in Fig. 5), so that in very hub-oriented scenarios (i.e. large networks with high fraction of controlled nodes), **u**_ref_ is nearly a standard basis vector, while in smaller networks wherein fewer nodes are controlled, **u**_ref_ is more homogenous.

**Figure 6:**
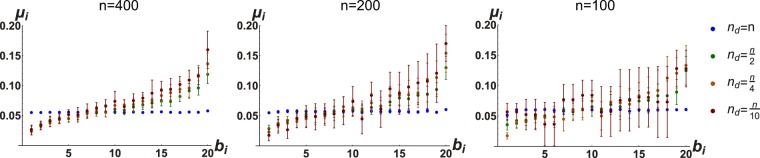
Same setup as in Fig. 5, but simulations are run for randomly connected (Erdös-Rènyi) networks (edge probability *p* = 0.5). Note the much smaller range of values on the vertical axis when compared with Fig. 5. Nodes of high degree (‘hubs’) are targeted, but to a lesser extent than for the scale-free networks. Skewness of the graphs is inversely related to *n*_d_; that is, it is less necessary to target hubs for more fully actuated networks, until at *n*_d_ = *n*, **u**_ref_ is essentially uniform.

For comparison’s sake, we ran simulations with randomly connected (Erdös-Rènyi (ER), with 0.5 edge probability) instead of scale-free networks. These networks were also undirected and rendered stable by the same method (described in Methods). We did use small (<0.1) positive edge weights, rather than unitary weights, for these networks to render their analysis more numerically tractable. We see in Fig. 6 that the optimal **u**_ref_ also tends to target nodes of higher degree in random ER networks, but to a much lesser extent than for scale-free networks. We hypothesize that this is because the degree distribution for scale-free networks is given by a power-law, which means there are many nodes of very low degree, and a few of very high degree. ER random networks have a binomial degree distribution, with more nodes of average degree and none of very high degree. Thus, it may be less crucial for the input to target the higher-degree nodes in ER random networks, simply because the higher-degree nodes are not *much* higher-degree nodes. In the ER random networks, we see a skewing of the values of **u**_ref_ which is inversely correlated with *n*_d_. That is, for less-actuated networks, the hubs tend to be more targeted, while for more fully-actuated networks, this targeting becomes less pronounced until at the limiting case (*n*_d_ = *n*), the entries of **u**_ref_ are all nearly identical.

### Information Spectra (of S_u_) are Sensitive to Network Parameterization

We now turn our attention to the problem of *comparing different networks* according to their information capacity, as quantified by ${ {\mathcal I} }_{{\bf{u}}}$. For this we examine the information capacity by varying **u**_ref_ in (21), where the intuitive strategy is to let ${{\bf{u}}}_{\ast }$ range over the eigenvectors of **S**_**u**_. Thus, a holistic characterization of ${\rm{tr}}({ {\mathcal I} }_{{\bf{u}}})$ is provided simply by the eigenvalue spectrum of **S**_**u**_ (recall that (21) takes on the value *λ*_*i*_, the *i*^th^ eigenvalue, when **u**_ref_ is the *i*^th^ eigenvector), heretofore termed the *information spectrum* of a network.

We obtained a distribution of information spectra for several network parametrizations. We here restricted our attention to steady state characterizations. Each distribution amounts to an empirical probability distribution of the eigenvalues of **S**_**u**_ over (random) network realizations. We assumed used zero-mean, unit-variance, uncorrelated noise (i.e. ${\mathbb{E}}[{{\bf{w}}}_{i}{{\bf{w}}}_{j}]={\mathbb{E}}[{{\bf{w}}}_{i}]{\mathbb{E}}[{{\bf{w}}}_{j}]=0\,\forall \,i$, $j\in \{1,\ldots ,n\}$, *i* ≠ *j* and ${\mathbb{E}}[{{\bf{w}}}_{i}{{\bf{w}}}_{i}]=1\,\forall \,i\in \{1,\ldots ,n\}$), though similar results were obtained for correlated noise.

**Figure 7:**
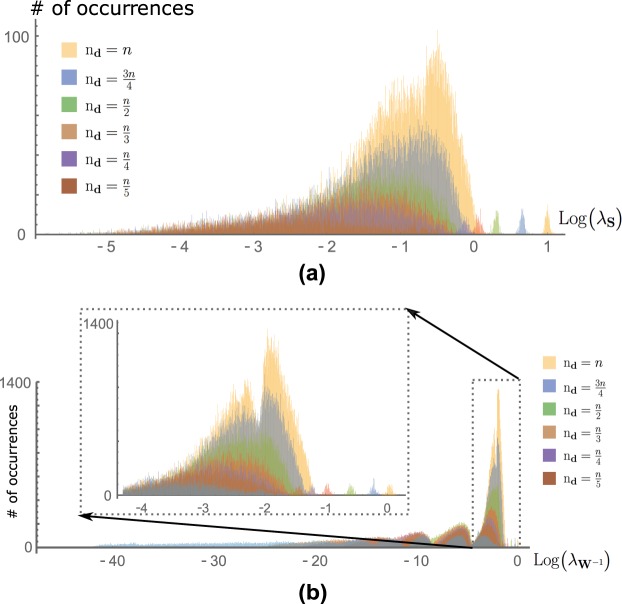
(**a**) Information spectra as function of number of actuated nodes (distributions aggregated over *n* = 100, 200, 300, 400). Spectra consist of a primary mode and a smaller secondary mode. (**b**) Spectra of the controllability gramian for different fractions of actuated nodes. As noted in previous work, these spectra display an increasing number of modes as *n*_d_ decreases. The principal mode is inset. Comparing to the information spectra in (**a**), we see that information spectra show marked similarity to first mode of control spectra, and both spectra reveal outlying, small modes corresponding to easiest (control) and most informative (information) directions.

Figure 7(a) depicts the information spectra for several fractions of actuated (driver) nodes (aggregates over several values of *n*). A first observation is the presence of a small, secondary mode to the right of the principal mode. This secondary mode reflects the presence of a few particularly salient inputs that most informatively correlate with all others. It is notable that this mode, which represents the largest eigenvalue of **S**_**u**_, systematically decreases with smaller values of *n*_d_. Certain intuition about these observations can be deduced from the rich body of work on spectra of random matrices. One such spectral characterization^28^ shows that the principal eigenvalue of the adjacency matrix (here denoted **A**) for undirected, binary scale-free networks (such as those used for our simulations, with the exception that the diagonal of our **A** is adjusted, as described in Methods, to ensure stability) approximates ${n}^{\frac{1}{4}}$, where *n* is the number of network nodes. Further, recent work^29^ has shown that this maximum eigenvalue, for weighted scale-free networks with expected degree distributions, varies monotonically with the maximum node degree. Maximum degree, in turn, increases dramatically as *n* increases, because of the preferential attachment-based network creation algorithm^30^. Thus we would expect the spectrum of **S**_**u**_, and in particular its principle eigenvalue, to depend on effective network size, which itself depends on *n*_d_ (see (11), and note the effect of **B** on (20)). This makes sense intuitively, as well: We would expect higher-dimensional input spaces to admit a richer set of encoded representations.

Further, as *n*_d_ decreases, the distribution of the main mode becomes broader and more entropic. No additional modes or ‘humps’ appear as *n*_d_ varies, a point we will return to shortly.

### Information Spectra are Related to the Controllability Gramian

As noted previously, the information spectrum is fundamentally time-varying (governed by the network dynamics, driven by the input in question). We were particularly interested in the relationship between the information spectrum and that of the controllability gramian matrix

$${\bf{W}}(t)=({\int }_{0}^{t}\,{{\rm{e}}}^{{\bf{A}}(t-\tau )}{\bf{B}}{{\bf{B}}}^{T}{{\rm{e}}}^{{{\bf{A}}}^{T}(t-\tau )}{\rm{d}}\tau ),$$

which also fundamentally characterizes the input-output relationship of a linear (networked) system. Indeed, it is well known that in the limit as $t\to \infty$, the gramian is exactly equivalent to Σ_**x**_, i.e., the denominator of ${ {\mathcal I} }_{{\bf{u}}}$. Thus, we sought to compare the information spectrum to that of **W**(∞).

The gramian matrix has been a pivotal entity in the analysis of linear systems and similarly modeled networks^31–33^, including certain types of brain networks^12,34^. Recent theoretical work^24^ has characterized the nature of the infinite-time gramian spectrum as a function of the number of driven nodes (*n*_d_). It is shown there that for small fractions of driven nodes the spectrum manifests a series of modes or ‘humps,’ over which eigenvalues are randomly distributed (over network realizations). As is well known in linear systems theory, the magnitude of a gramian eigenvalue determines the minimum input energy needed to reach the unit hypersphere in the direction of its associated eigenvector. Thus, the principal mode of the gramian spectrum describes those directions that are ‘easiest’ to induce.

Figure 7(b) depicts the gramian spectrum for the same networks as in Fig. 7(a) (i.e., with varying fraction of actuated nodes). The aforementioned modes are readily evident. What is notable from this figure is the correspondence between the information spectra to the two rightmost modes of the gramian spectrum (that is, the principal mode and the much smaller mode at far right). In interpreting this result, it is important to note that the information and gramian spectra are of different dimensions $({{\bf{W}}}^{-1}({t}_{f})\in {{\mathbb{R}}}^{n{\rm{x}}n}$ while ${{\bf{S}}}_{{\bf{u}}}\in {{\mathbb{R}}}^{m{\rm{x}}m})$. This is because the information spectrum captures only constant inputs, thus for a fixed time the state is restricted to an *m*-dimensional subspace. In this sense, we postulate that the principal mode of the gramian spectrum corresponds not simply to the ‘easiest’ to reach directions, but also those associated with constant (*m*-dimensional) inputs.

Let us now seek to understand this numerical correspondence between control and information, shown in Fig. 7, at a conceptual level. What does it *mean* that the easiest directions of control (quantified by the largest eigenvalues of **W**^−1^) and network information (quantified by ${ {\mathcal I} }_{{\bf{u}}}$) show such similarity? We hypothesize that this correspondence may be indicative of an underlying link between controllability metrics and information-based analyses, generally. Indeed, this is not a novel idea; the mathematical basis for this link has been explored^35,36^ in contexts different, but related, to ours. We can summarize the essence of these discussions, as it relates to our formulation, simply by noting that ${ {\mathcal I} }_{{\bf{u}}}$ depends fundamentally on a derivative of the state (to be more precise, an inner product of two states) *with respect to* **u**. Thus, when system dynamics are such that incremental changes made to **u** result in large changes to the state, informational value is increased. This information is, to some extent, a measure of network sensitivity to its inputs, and sensitivity to inputs is, of course, exactly what controllability analysis quantifies.

## Discussion

We developed an analysis to quantify the amount of information about an input **u** that can be gleaned from the contrast/correlation between its induced state **x**_**u**_ and a reference or background state **x**_ref_. Our analysis shows that there exists an optimally informative **x**_ref_ in this context. This theoretical result reinforces intuition about how proper choice of a contrasting background might enable more rapid decoding and subsequent processing of input stimuli. We showed that the orientation of **x**_ref_ relative to the principal axis of noise covariance decreased monotonically with increasing fraction of nodes actuated and that this separation also decreased over time, but to an extent limited by *n*_d_ This *dynamical* relationship between the informational optimum and the noise covariance is complementary to results based on static models^9^.

We expanded our inquiry to examine the **u**_ref_ which would give rise to **x**_ref_. We found that the optimal **u**_ref_ tends to target network hubs, but in a way which varies consistently with number of nodes driven *n*_d_ (See Fig. 5). We then derived an information spectrum that characterizes the full encoding capacity (in terms of inner product readout) of inputs. We showed that this spectrum has nuanced dependency on network size and fraction of driven nodes, with the presence of a low-dimensional set of inputs to which networks appear particularly well-tuned. Further, we reconciled the information encoding of a network with its control-theoretic properties, which characterize how the ‘energy’ of an input allow for the state space to be traversed. Our results suggest that inputs that produce ‘easy’ state excursions–recall that these inputs are postulated to be constant or near-constant (see Section)–are also those that are well-encoded.

It may reasonably be asked why we have chosen inputs to be constant in the overall paradigm. At a conceptual level, our information analysis is fundamentally predicated on the derivative $\frac{\partial \langle {\bf{x}},{{\bf{x}}}_{{\rm{ref}}}\rangle }{\partial {\bf{u}}}$. That is, we seek to quantify the extent to which changes in the projection of system state **x** onto background **x**_ref_ reflect incremental changes in **u**. In the case of a constant **u**, this is readily interpreted – it quantifies the ability to deduce changes in the input composition. However, interpretability is more problematic for a time-varying **u**(*t*). What does it mean to make an incremental change in the *function* **u**(*t*)? Is the relevant change spatial (composition) or temporal? In this sense, because we are dealing with a variational problem in infinite-dimensional function space, intuition is difficult.

This argument can be seen mathematically. Examining (3), we see that taking a derivative with respect to **u**(*t*) presents us with the task of taking the derivative of one function of *t* (${\mathbb{E}}[\langle {\bf{x}}(t),{{\bf{x}}}_{{\rm{ref}}}\rangle ]$) with respect to another function (**u**(*t*)). Thus, ${ {\mathcal I} }_{{\bf{u}}}$ would become dependent on **u**′(*t*). But we conduct our analysis with respect to the objective of *learning* about **u** from a ‘readout’ of only the projection of **x**(*t*) onto **x**_ref_. To assume a knowledge of the time derivative of **u**(*t*) changes the setup completely. One way around this dilemma would be to project **u**(*t*) onto a set of orthogonal basis functions (a Fourier basis, for example). If we denote a vector of basis functions (truncated so as not to be infinite) as **h**(*t*), we can approximate (almost) any **u**(*t*) by **Uh**(*t*), where **U** is a constant projection, or coefficient, matrix. Then, ${ {\mathcal I} }_{{\bf{u}}}$ becomes linear in **U** and the basic formulation is preserved, with the change that instead of seeking to infer constant input **u** via the state projection, we seek to infer coefficient matrix **U**. A thorough treatment of this idea will be given in future work.

Having highlighted the results from the exploration of ${ {\mathcal I} }_{{\bf{u}}}$, let us take a slightly higher-level look at the information processing which ${ {\mathcal I} }_{{\bf{u}}}$ quantifies. Considering the inner product as the ‘readout’ (which forms the basis of information measure ${ {\mathcal I} }_{{\bf{u}}}$) is intuitive since it measures correlation/contrast between two competing representations of a stimulus. In this sense, it is a highly condensed representation of potentially high-dimensional stimuli. However, it is far from clear whether a network itself could accomplish this readout, and whether this is in fact a reasonable strategy for actual information processing tasks such as input classification. The linearity of the model considered is certainly a limiting factor in this regard.

Nonetheless, we believe our results highlight an interesting direction toward analyzing not simply the structural aspects of networks, but also their dynamics and ultimately their functionality. It is straightforward to envision generalizing our framework to examine other network topologies, dynamical nonlinearities and wider time-scales, as well as alternative information metrics. These types of analyses can shed light on the functional advantages of biological networks (e.g., those in the brain) and/or principles for guiding the design of engineered systems.

## Methods

### Derivation of ${\mathcal I}$_u_

Proceeding from (7), we make use of the fact that ${{\rm{\Sigma }}}_{\langle {\bf{x}},{{\bf{x}}}_{{\rm{ref}}}\rangle }$ is a scalar (being the variance of a scalar inner product), so that

22 $${ {\mathcal I} }_{{\bf{u}}}=\frac{{{\bf{B}}}^{T}{{\rm{\Gamma }}}^{T}{{\bf{x}}}_{{\rm{ref}}}{{\bf{x}}}_{{\rm{ref}}}^{T}{\rm{\Gamma }}{\bf{B}}}{{{\rm{\Sigma }}}_{\langle {\bf{x}},{{\bf{x}}}_{{\rm{ref}}}\rangle }}$$

In seeking a holistic assessment of the matrix ${ {\mathcal I} }_{{\bf{u}}}$, we employ the trace, which is the summed component-wise variance in our estimation of **u**. Since ${ {\mathcal I} }_{{\bf{u}}}$ is an outer product of two vectors we may express its trace as their inner product

23 $${\rm{tr}}({ {\mathcal I} }_{{\bf{u}}})=\frac{{{\bf{x}}}_{{\rm{ref}}}^{T}{\rm{\Gamma }}{\bf{B}}{{\bf{B}}}^{T}{{\rm{\Gamma }}}^{T}{{\bf{x}}}_{{\rm{ref}}}}{{{\rm{\Sigma }}}_{\langle {\bf{x}},{{\bf{x}}}_{{\rm{ref}}}\rangle }}$$

We now examine the inner product variance ${{\rm{\Sigma }}}_{\langle {\bf{x}},{{\bf{x}}}_{{\rm{ref}}}\rangle }$. It is straightforward to obtain

24 $${{\rm{\Sigma }}}_{\langle {\bf{x}},{{\bf{x}}}_{{\rm{ref}}}\rangle }={\mathbb{E}}[{\langle {\bf{x}},{{\bf{x}}}_{{\rm{ref}}}\rangle }^{2}]-{({\mathbb{E}}[\langle {\bf{x}},{{\bf{x}}}_{{\rm{ref}}}\rangle ])}^{2}$$

25 $$=\,{\mathbb{E}}[({{\bf{x}}}^{T}{{\bf{x}}}_{{\rm{ref}}}{)}^{T}({{\bf{x}}}^{T}{{\bf{x}}}_{{\rm{ref}}})]-{({\mathbb{E}}{[{\bf{x}}]}^{T}{\mathbb{E}}[{{\bf{x}}}_{{\rm{ref}}}])}^{2}$$

26 $$=\,{{\bf{x}}}_{{\rm{ref}}}^{T}{\mathbb{E}}[{\bf{x}}{{\bf{x}}}^{T}]{{\bf{x}}}_{{\rm{ref}}}-{({{\bf{u}}}^{T}{{\bf{B}}}^{T}{{\rm{\Gamma }}}^{T}{{\bf{x}}}_{{\rm{ref}}})}^{2}$$

Note that ${\mathbb{E}}[{\bf{x}}{{\bf{x}}}^{T}]$ is the correlation matrix of **x**, so that

27 $${{\rm{\Sigma }}}_{{\bf{x}}}={\mathbb{E}}[({\bf{x}}-{\mathbb{E}}[{\bf{x}}])\,{({\bf{x}}-{\mathbb{E}}[{\bf{x}}])}^{T}]$$

28 $$=\,{\mathbb{E}}[{\bf{x}}{{\bf{x}}}^{T}]-{\rm{\Gamma }}{\bf{B}}{\bf{u}}{{\bf{u}}}^{T}{{\bf{B}}}^{T}{{\rm{\Gamma }}}^{T}$$

Therefore

29 $${\mathbb{E}}[{\bf{x}}{{\bf{x}}}^{T}]={{\rm{\Sigma }}}_{{\bf{x}}}+{\rm{\Gamma }}{\bf{B}}{\bf{u}}{{\bf{u}}}^{T}{{\bf{B}}}^{T}{{\rm{\Gamma }}}^{T}$$

and combining (29) with (26) we have

30 $$\begin{array}{rcl}{{\rm{\Sigma }}}_{\langle {\bf{x}},{{\bf{x}}}_{{\rm{ref}}}\rangle } & = & {{\bf{x}}}_{{\rm{ref}}}^{T}({{\rm{\Sigma }}}_{{\bf{x}}}+{\rm{\Gamma }}{\bf{B}}{\bf{u}}{{\bf{u}}}^{T}{{\bf{B}}}^{T}{{\rm{\Gamma }}}^{T}){{\bf{x}}}_{{\rm{ref}}}\\  &  & -\,{({{\bf{u}}}^{T}{{\bf{B}}}^{T}{{\rm{\Gamma }}}^{T}{{\bf{x}}}_{{\rm{ref}}})}^{2}\end{array}$$

31 $$=\,{{\bf{x}}}_{{\rm{ref}}}^{T}{{\rm{\Sigma }}}_{{\bf{x}}}{{\bf{x}}}_{{\rm{ref}}}$$

Thus, plugging (31) into (22) we have the Fisher information matrix

32 $${ {\mathcal I} }_{{\bf{u}}}=\frac{{{\bf{B}}}^{T}{{\rm{\Gamma }}}^{T}{{\bf{x}}}_{{\rm{ref}}}{{\bf{x}}}_{{\rm{ref}}}^{T}{\rm{\Gamma }}{\bf{B}}}{{{\bf{x}}}_{{\rm{ref}}}^{T}{{\rm{\Sigma }}}_{{\bf{x}}}{{\bf{x}}}_{{\rm{ref}}}}$$

as given in the main body of the text.

### Network parameterization and simulations

To ensure stability, it is sufficient^24^ to ensure that, $\forall \,i\in \{1,\ldots ,n\}$, the *i*^th^ diagonal element of binary adjacency matrix **A** is at least as negative as the sum of the non-diagonal elements in row *i*. That is, ${\sum }_{j\ne i}\,{{\bf{A}}}_{i,j}+{{\bf{A}}}_{i,i} < 0$. Accordingly, in constructing networks, we first created a scale-free degree distribution and then formed a corresponding random graph, thus prescribing adjacency matrix **A**. Next we simply assigned ${{\bf{A}}}_{i,i}=-\,({\sum }_{j\ne i}\,{{\bf{A}}}_{i,j}+{\delta }_{i})$, where each *δ*_*i*_ was picked at random from (0, 1).

Creation of these adjacency matrices and the **B** matrices, as well as the calculations of optimally contrasting background state **x**_ref_ and reference stimulus **u**_ref_, with the associated statistical analyses, were performed using *Mathematica*, with the exception of the calculations of controllability gramians, which were done by exporting these matrices to MATLAB, and using the lyap() command.
